# Retraction Note: In silico and in vitro assessment of TP53, ATM, RAD51, and BAX genes in gastric cancer and their contribution to radiotherapy resistance

**DOI:** 10.1186/s41065-026-00753-z

**Published:** 2026-10-10

**Authors:** Junwei Zhang, Pengtao He

**Affiliations:** 1 Department of Radiotherapy, Chang’an Hospital, Xi’an, 71000 China; 2 Department of Oncology Geriatrics, Xi’an Fengcheng Hospital, Xi’an, 710000 China

**Retraction Note: Hereditas 162, 125 (2025)**

**https://doi.org/10.1186/s41065-025-00496-3**

The Editor-in-Chief has retracted this article. After publication, concerns were raised regarding the in vitro images presented in Figs. 8 and 9 of this article, where the patterns of cells and colonies do not appear consistent with natural cell growth and proliferation. Additionally, the uncut western blot gel presented in the Supplementary Material appears to show contradictory results, with three proteins probed in adjacent lanes with different results. It has also been noted that a number of references in this article do not appear to support the statements preceding the citations, and Fig. 1A and B appear to present contradictory data.

The Editor-in-Chief therefore no longer has confidence in the presented data.

The authors have not responded to correspondence regarding this retraction.

